# Constraints on antisymmetric tensor fields from Bhabha scattering

**DOI:** 10.1140/epjc/s10052-021-09915-x

**Published:** 2021-12-18

**Authors:** Siddharth Tiwary, Rainer Dick

**Affiliations:** 1 Department of Physics and Engineering Physics, University of Saskatchewan, 116 Science Place, Saskatoon, SK S7N 5E2 Canada; 2 Department of Physics, Indian Institute of Technology Bombay, Powai, Mumbai, 400076 India

## Abstract

Antisymmetric tensor fields are a compelling prediction of string theory. This makes them an interesting target for particle physics because antisymmetric tensors may couple to electromagnetic dipole moments, thus opening a possible discovery opportunity for string theory. The strongest constraints on electromagnetic dipole couplings would arise from couplings to electrons, where these couplings would contribute to Møller and Bhabha scattering. Previous measurements of Bhabha scattering constrain the couplings to ${\tilde{M}}_e m_C>7.1\times 10^4\,{\mathrm {GeV}}^2$, where $m_C$ is the mass of the antisymmetric tensor field and ${\tilde{M}}_e$ is an effective mass scale appearing in the electromagnetic dipole coupling.

## Introduction

String theory has become an increasingly complex and compelling framework for particle physics beyond the Standard Model. Discovering string signatures through extra dimensions, $Z'$ bosons, Regge excitations, supersymmetry or string moduli has become a significant science driver for the development of next-generation accelerators [1, 2], and the advent of low-scale string models [3] has increased the potential accessibility of all these signatures. While supersymmetry and Kaluza–Klein modes were traditionally studied as possible indicators for the correctness of string theory for about half a century now, the prospects of low-scale string theory added the direct detection of string excitations as a further possibility [4–14]. String signatures from Kaluza–Klein modes and $Z'$ bosons at the LHC have also been studied [15, 16].

The discovery of Regge excitations would constitute a smoking gun signature for string theory. However, the discovery of antisymmetric tensor fields would also provide a very strong indication for the correctness of string theory. Antisymmetric tensor excitations appear in the massless sector of closed strings and throughout the excited levels of closed and open strings, and Kalb and Ramond have pointed out that antisymmetric tensor fields can also mediate gauge interactions between strings [17]. Quantization of antisymmetric tensors leaves only a single externally propagating transverse polarization state [18]. However, the couplings of the tensor are still restricted by Lorentz invariance and the non-physical transverse polarization states contribute as virtual states to scattering amplitudes. This makes antisymmetric tensor fields another interesting target for exploration of possible low-energy signals of string theory.

Both standard string theory and the Kalb–Ramond proposal include couplings of antisymmetric tensor fields ${\mathcal {C}}_{\mu \nu }(x)$ to string world sheets ${\mathcal {S}}$: $X^\mu (\tau ,\sigma )\equiv X^\mu (\sigma ^1,\sigma ^2)$ in the form

1 $$\begin{aligned} S_{X{\mathcal {C}}}= & {} \mu _s\int _{{\mathcal {S}}}\!{\mathcal {C}} \nonumber \\= & {} \frac{\mu _s}{2}\int \!d^2\sigma \left( {\dot{X}}^\mu X'^\nu -{\dot{X}}^\nu X'^\mu \right) {\mathcal {C}}_{\mu \nu }(X). \end{aligned}$$

Since we are interested in low energy string phenomenology we avoid the usual designation $B_{\mu \nu }$ for the antisymmetric tensor fields to avoid confusion with the $U_Y(1)$ field strength in the Standard Model. We also include a string charge $\mu _s$ with mass dimension 1 to have canonical mass dimension 1 for the antisymmetric tensor field, such that the kinetic term for the Kalb–Ramond field strength ${\mathcal {C}}_{\mu \nu \rho }= \partial _\mu {\mathcal {C}}_{\nu \rho }+\partial _\nu {\mathcal {C}}_{\rho \mu }+\partial _\rho {\mathcal {C}}_{\mu \nu }$ in four spacetime dimensions can be written as ${\mathcal {L}}_{d{\mathcal {C}}}=-\,{\mathcal {C}}^{\mu \nu \rho }{\mathcal {C}}_{\mu \nu \rho }/6$. The Kalb–Ramond picture of gauge interactions between strings also includes dimensionless string boundary charges $g_s$ and a vector field ${\mathcal {B}}_\mu$ which couples to the boundary $\partial {\mathcal {S}}$ of open string world sheets,

2 $$\begin{aligned} S_{X{\mathcal {B}}}=g_s\int _{\partial {\mathcal {S}}}\!{\mathcal {B}} =g_s\int \! d\tau \left[ {\dot{X}}^\mu {\mathcal {B}}_{\mu }(X)\right] _{\sigma =0}^{\sigma =\ell }. \end{aligned}$$

This is appealing, because it yields a mass $m_C=\mu _s/\sqrt{2}g_s$ for the Kalb–Ramond field in the four-dimensional spacetime action,

3 $$\begin{aligned} {\mathcal {L}}= & {} -\,\frac{1}{6}{\mathcal {C}}^{\mu \nu \rho }{\mathcal {C}}_{\mu \nu \rho } -\frac{1}{4}{\mathcal {B}}^{\mu \nu }{\mathcal {B}}_{\mu \nu } +\frac{\mu _s}{2g_s}{\mathcal {C}}^{\mu \nu }{\mathcal {B}}_{\mu \nu } \nonumber \\&-\,\frac{\mu _s^2}{4g_s^2}{\mathcal {C}}^{\mu \nu }{\mathcal {C}}_{\mu \nu } +j^{\mu \nu }{\mathcal {C}}_{\mu \nu }+j^\mu {\mathcal {B}}_\mu , \end{aligned}$$

without breaking the KR gauge symmetries

4 $$\begin{aligned}&{\mathcal {C}}_{\mu \nu }\rightarrow {\mathcal {C}}_{\mu \nu }+\partial _\mu f_\nu -\partial _\nu f_\mu , \end{aligned}$$

5 $$\begin{aligned}&{\mathcal {B}}_\mu \rightarrow {\mathcal {B}}_\mu +(\mu _s/g_s)f_\mu +\partial _\mu f. \end{aligned}$$

The string currents in (3) are from (1,2)

6 $$\begin{aligned} j^{\mu \nu }(x)= & {} \frac{\mu _s}{2}\int \! d\tau \!\int _0^\ell \! d\sigma \left[ {\dot{X}}^\mu (\tau ,\sigma ) X'^\nu (\tau ,\sigma ) \right. \nonumber \\&-\left. {\dot{X}}^\nu (\tau ,\sigma ) X'^\mu (\tau ,\sigma )\right] \delta (x-X(\tau ,\sigma )), \end{aligned}$$

7 $$\begin{aligned} j^{\mu }(x)= & {} g_s\!\int \! d\tau \left[ {\dot{X}}^\mu (\tau ,\sigma ) \delta (x-X(\tau ,\sigma ))\right] _{\sigma =0}^{\sigma =\ell }. \end{aligned}$$

They satisfy the consistency conditions

8 $$\begin{aligned} \partial _\mu j^{\mu \nu }(x)=(\mu _s/2g_s)j^\nu (x),\quad \partial _\mu j^{\mu }(x)=0. \end{aligned}$$

The gauge invariant field^1^

11 $$\begin{aligned} C_{\mu \nu }={\mathcal {C}}_{\mu \nu }-\frac{g_s}{\mu _s}{\mathcal {B}}_{\mu \nu } \end{aligned}$$

satisfies the equations of motion

12 $$\begin{aligned} \partial _\mu C^{\mu \nu \rho }(x)-\frac{\mu _s^2}{2g_s^2} C^{\nu \rho }(x)=-\,j^{\nu \rho }(x) \end{aligned}$$

and

13 $$\begin{aligned} \partial _\mu C^{\mu \nu }(x)=\frac{g_s}{\mu _s}j^{\nu }(x). \end{aligned}$$

These equations imply that the Kalb–Ramond field in the interaction picture is a transverse massive antisymmetric tensor field with mode expansion

9 $$\begin{aligned} C_{\mu \nu }(x)= & {} \int \!\frac{d^3{\varvec{k}}}{4\sqrt{2\pi ^3E({\varvec{k}})}}\,\epsilon _{\alpha \beta \gamma } \epsilon ^{(\beta )}_\mu ({\varvec{k}})\epsilon ^{(\gamma )}_\nu ({\varvec{k}}) \nonumber \\&\times [a^{(\alpha )}({\varvec{k}})\exp ({\mathrm {i}}k\cdot x) +\,a^{(\alpha )+}({\varvec{k}})\exp (-\,{\mathrm {i}}k\cdot x)].\nonumber \\ \end{aligned}$$

We choose polarization vectors $\epsilon ^{(\alpha )}_\mu ({\varvec{k}})$ such that for $\alpha \in \{1,2\}$

14 $$\begin{aligned} k\cdot \epsilon ^{(\alpha )}({\varvec{k}})={\varvec{k}}\cdot {\varvec{\epsilon }}^{(\alpha )}({\varvec{k}})=0, \end{aligned}$$

whereas $$\epsilon _0^{(3)}({\varvec{k}})\ne 0$$,

15 $$\begin{aligned} k\cdot \epsilon ^{(3)}({\varvec{k}})=0\ne {\varvec{k}}\cdot {\varvec{\epsilon }}^{(3)}({\varvec{k}}). \end{aligned}$$

Comparison with the Kalb–Ramond field in Coulomb gauge [20] shows that the single completely transverse physical polarization state is given by $\epsilon ^{(1)}_\mu ({\varvec{k}})\epsilon ^{(2)}_\nu ({\varvec{k}})-\epsilon ^{(2)}_\mu ({\varvec{k}})\epsilon ^{(1)}_\nu ({\varvec{k}})$, whereas the two (spatially longitudinal but 4d transverse) polarizations $[{\varvec{\epsilon }}^{(\alpha )}({\varvec{k}})\otimes {\varvec{\epsilon }}^{(3)}({\varvec{k}}) -{\varvec{\epsilon }}^{(3)}({\varvec{k}})\otimes {\varvec{\epsilon }}^{(\alpha )}({\varvec{k}})]_{\alpha \in \{1,2\}}$ are unphysical. Therefore only $a^{(3)+}({\varvec{k}})$ generates external physical states for the Kalb–Ramond field, but the other transverse modes also contribute to virtual Kalb–Ramond exchange.

The normalization in (13) was chosen such that the canonical commutation relation

16 $$\begin{aligned}&[C_{\mu \nu }({\varvec{x}},t),\partial _0 C_{\rho \sigma }({\varvec{x}}',t)] ={{\mathrm {i}}}\delta ^\perp _{\mu \nu \rho \sigma }({\varvec{x}}-{\varvec{x}}') \nonumber \\&\quad ={{\mathrm {i}}}\int \!\frac{d^3{\varvec{k}}}{(2\pi )^3}\exp [{{\mathrm {i}}}{\varvec{k}}\cdot ({\varvec{x}}-{\varvec{x}}')] \nonumber \\&\qquad \times \frac{1}{2} [P^\perp _{\mu \rho }({\varvec{k}})P^\perp _{\nu \sigma }({\varvec{k}})-P^\perp _{\mu \sigma }({\varvec{k}})P^\perp _{\nu \rho }({\varvec{k}})], \end{aligned}$$

17 $$\begin{aligned}&P^\perp _{\mu \rho }({\varvec{k}})=\epsilon ^{(\alpha )}_\mu ({\varvec{k}})\epsilon ^{(\alpha )}_\rho ({\varvec{k}}), \end{aligned}$$

yields

18 $$\begin{aligned}{}[a^{(\alpha )}({\varvec{k}}),a^{(\beta )+}({\varvec{k}}')]=\delta _{\alpha \beta }\delta ({\varvec{k}}-{\varvec{k}}'). \end{aligned}$$

The propagator for the Kalb–Ramond field is

19 $$\begin{aligned}&G_{\mu \nu ,\kappa \lambda }(x-x')={{\mathrm {i}}} {\varvec{\langle }} 0{\varvec{|}}{\mathrm {T}}\,C_{\mu \nu }(x)C_{\kappa \lambda }(x'){\varvec{|}}0{\varvec{\rangle }} \nonumber \\&\quad =\int \!\frac{d^4k}{32\pi ^4}\, \frac{\exp [{{\mathrm {i}}}k\cdot (x-x')]}{k^2+m_C^2-{{\mathrm {i}}}\epsilon } \nonumber \\&\qquad \times [P^\perp _{\mu \kappa }({\varvec{k}})P^\perp _{\nu \lambda }({\varvec{k}})-P^\perp _{\mu \lambda }({\varvec{k}})P^\perp _{\nu \kappa }({\varvec{k}})]. \end{aligned}$$

This fits into canonical string theory if we assume that antisymmetric tensors are spacetime manifestations of tensor excitations of strings. Another, more speculative interpretation of (3) would suggest that strings and quantum fields may co-exist, such that strings define a genuine extension of quantum field theory without encompassing the quantum field theories of particle physics as a mere low-energy effective description. Strings are then classical objects (from the target space quantum field theory perspective) which carry two-dimensional quantum field theories for their embeddings, whereas point particles are quantized in the standard way. In such a framework, antisymmetric tensors would act as mediators between point particles and strings.

Either way, antisymmetric tensor fields are unavoidable in string theory and we should study their possible signatures in particle physics experiments.

Antisymmetric tensors can couple in particular to electromagnetic dipole moments, thus contributing to Møller and Bhabha scattering. This is particularly relevant for upcoming or proposed lepton colliders [1, 2, 21, 22], because the well-defined initial state in the scattering events facilitates the search for deviations from Standard Model scattering cross sections. Existing data on Bhabha scattering from previous ${\mathrm {e}}^+{\mathrm {e}}^-$ collider experiments already limit deviations from Standard Model cross sections, and here we report constraints on antisymmetric tensors using published data from TASSO [23, 24], PLUTO [25], MAC [26], TOPAZ [27] and OPAL [28].

Although our primary interest is on collider-based constraints and prospects for antisymmetric tensor fields as harbingers of string theory, we note in passing that an antisymmetric tensor might also lend itself as a dark matter candidate due to its electroweak singlet properties. Within the coupling model (20) that we investigate for particle physics implications, this possibility is excluded from the requirement of longevity: Mass values in the MeV range or below for the antisymmetric tensor field are compatible with a lifetime of order $10^{18}$ s if the coupling to neutrinos satisfies $v_h/M_n\lesssim 10^{-16}$ (assuming $a_M^2+a_e^2\simeq 1$), i.e. if the coupling scale $M_n$ is of order of the reduced Planck mass. However, light antisymmetric tensors with Standard Model couplings of the form (20) would have revealed their existence through resonances in scattering experiments, whereas heavy antisymmetric tensors decay too fast to serve as dark matter. On the other hand, antisymmetric tensor fields could serve as messengers into a dark sector [19], but we will focus on their corrections to Møller and Bhabha scattering in the following.

We discuss the contribution from antisymmetric tensors to Møller and Bhabha scattering in Sect. 2. Constraints on the antisymmetric tensor mass and the coupling to electrons are reported in Sect. 3. Section 4 summarizes our conclusions.

## Bhabha scattering through Kalb–Ramond exchange

The KR gauge invariant tensor $C_{\mu \nu }$ can have $SU_w(2)\times U_Y(1)$ invariant couplings to Standard Model fermions through interaction terms

20 $$\begin{aligned} {\mathcal {L}}_I= & {} -\,\frac{1}{M_e}{\overline{\Psi }}\cdot H S^{\mu \nu }(a_m+{\mathrm {i}}a_e\gamma _5) \frac{1+\gamma _5}{\sqrt{2}}\psi _e C_{\mu \nu } \nonumber \\&-\,\frac{1}{M_n}{\overline{\Psi }}\cdot {\tilde{H}} S^{\mu \nu }(a_m+{\mathrm {i}}a_e\gamma _5) \frac{1+\gamma _5}{\sqrt{2}}\psi _n C_{\mu \nu } \nonumber \\&+\,{\mathrm {h.c.}} \end{aligned}$$

Here

21 $$\begin{aligned} \Psi =\left( \begin{array}{c} \psi _n\\ \psi _e\\ \end{array}\right) \end{aligned}$$

are $SU_w(2)$ spinors with neutrino/up-type upper fields and electron/down-type lower fields and

22 $$\begin{aligned} {\underline{H}}=\left( \begin{array}{c} H^+\\ H^0\\ \end{array}\right) ,\quad \tilde{{\underline{H}}}={\underline{\epsilon }}\cdot {\underline{H}}^* =\left( \begin{array}{c} \,\,\,\, H^{0,*}\\ -H^{+,*}\\ \end{array}\right) \end{aligned}$$

is the Higgs doublet. We use the spinor representation of the Lorentz generators,

23 $$\begin{aligned} S^{\mu \nu }=\frac{1}{2}\sigma ^{\mu \nu }=\frac{{\mathrm {i}}}{4}[\gamma ^\mu ,\gamma ^\nu ], \end{aligned}$$

in the dipole operators. The leading order coupling to the Standard Model fermions from (20) is then

24 $$\begin{aligned} {\mathcal {L}}_{eC}=-\,\frac{v_h}{M_e}{\overline{\psi }}_e S^{\mu \nu }(a_m+{\mathrm {i}}a_e\gamma _5) \psi _e C_{\mu \nu }, \end{aligned}$$

with the Higgs expectation value $v_h=\sqrt{2}\langle H^0\rangle$.

We assume unbroken KR gauge symmetry and therefore focus on the gauge invariant couplings (20) of the KR fields to Standard Model fermions. Any mixing of a string boundary gauge field ${\mathcal {B}}_\mu$ with Standard Model gauge fields, or promotion to a $Z'$ through a mass term, would break the KR gauge symmetry. These would then enhance the pool of string-motivated vector fields [16, 29] which could also contribute to $g-2$ values for Standard Model leptons [30–32]. However, here we are content with the observation that breaking of KR gauge symmetry would provide further motivation and relevance for the study of massive vector fields from string theory.

Absence of a resonance from antisymmetric tensor exchange in Bhabha scattering up to the highest LEP energies tells us that $m_C>209$ GeV. Furthermore, we have no reason to expect a fundamental antisymmetric tensor field from string theory to be hadrophobic, and absence of Beyond the Standard Model resonances up to the highest energies probed in hadronic collisions indicates $m_C>1$ TeV [33]. We will therefore analyze the coupling (24) under the assumption $\sqrt{s}\ll m_c$, since the highest collision energy used here is $\sqrt{s}=136.23$ GeV [28].

Exchange of virtual Kalb–Ramond tensor particles through the coupling (24) yields *t* and *u* channel contributions to Møller scattering and *s* and *t* channel contributions to Bhabha scattering. In order $\alpha _S v_h^2/M_e^2$, this shifts the corresponding cross sections at energy $\sqrt{s}$ by^2^

25 $$\begin{aligned} \left. \frac{d\sigma }{d\Omega }\right| _{v_h^2/M_e^{2}}= & {} \frac{\pi ^2}{4}s \left( {\mathcal {M}}^{(\gamma ,Z)+}{\mathcal {M}}^{(C)}+{\mathcal {M}}^{(C)+}{\mathcal {M}}^{(\gamma ,Z)}\right) ,\nonumber \\ \end{aligned}$$

where ${\mathcal {M}}^{(\gamma ,Z)}$ are the Møller or Bhabha scattering amplitudes through photon and *Z* exchange and ${\mathcal {M}}^{(C)}$ are the corresponding amplitudes from Kalb–Ramond exchange. Here we use a normalization of scattering amplitudes such that the scattering matrix with incoming 4-momentum $P_i$ and final 4-momentum $P_f$ is

26 $$\begin{aligned} S_{fi}=-\,{\mathrm {i}}{\mathcal {M}}_{fi}\delta (P_f-P_i). \end{aligned}$$

We imply summation/averaging $(1/4)\sum _{s'_2,s'_s}\sum _{s_1,s_2}$ for the outgoing/incoming spin orientations in the product of scattering amplitudes. Corrections for spin-polarized Møller scattering will be of interest for the upcoming MOLLER experiment.

The amplitude for Møller scattering ${\varvec{|}}{\varvec{k}}_1,\sigma _1;{\varvec{k}}_2,\sigma _2{\varvec{\rangle }}\rightarrow {\varvec{|}}{\varvec{p}}_1,s_1;{\varvec{p}}_2,s_2{\varvec{\rangle }}$ through Kalb–Ramond exchange is then

$$\begin{aligned}&{\mathcal {M}}^{(C)}_{--}=-\,\frac{v_h^2}{16\pi ^2 M_e^2 \sqrt{E({\varvec{p}}_1)E({\varvec{p}}_2)E({\varvec{k}}_1)E({\varvec{k}}_2)}} \\&\times \left( \frac{{\overline{u}}({\varvec{p}}_1,s_1)\Gamma ^{\mu \nu }u({\varvec{k}}_1,\sigma _1) {\overline{u}}({\varvec{p}}_2,s_2)\Gamma ^{\kappa \lambda }u({\varvec{k}}_2,\sigma _2)}{ (k_1-p_1)^2+m_C^2-{\mathrm {i}}\epsilon } \right. \\&\times \left[ P^{\perp }_{\mu \kappa }({\varvec{k}})P^{\perp }_{\nu \lambda }({\varvec{k}})\right] _{{\varvec{k}}={\varvec{k}}_1-{\varvec{p}}_1} -\left[ P^{\perp }_{\mu \kappa }({\varvec{k}})P^{\perp }_{\nu \lambda }({\varvec{k}})\right] _{{\varvec{k}}={\varvec{k}}_1-{\varvec{p}}_2} \\&\times \left. \frac{{\overline{u}}({\varvec{p}}_2,s_2)\Gamma ^{\mu \nu }u({\varvec{k}}_1,\sigma _1) {\overline{u}}({\varvec{p}}_1,s_1)\Gamma ^{\kappa \lambda }u({\varvec{k}}_2,\sigma _2)}{ (k_1-p_2)^2+m_C^2-{\mathrm {i}}\epsilon } \right) , \end{aligned}$$

where

27 $$\begin{aligned} \Gamma ^{\mu \nu }=S^{\mu \nu }(a_m+{\mathrm {i}}a_e\gamma _5). \end{aligned}$$

The amplitude for Bhabha scattering through Kalb–Ramond exchange is

$$\begin{aligned}&{\mathcal {M}}^{(C)}_{-+}=-\,\frac{v_h^2}{ 16\pi ^2 M_e^2\sqrt{E({\varvec{p}}_1)E({\varvec{p}}_2)E({\varvec{k}}_1)E({\varvec{k}}_2)}}\\&\times \left( \frac{{\overline{u}}({\varvec{p}}_1,s_1)\Gamma ^{\mu \nu } v({\varvec{p}}_2,s_2) {\overline{v}}({\varvec{k}}_2,\sigma _2)\Gamma ^{\kappa \lambda } u({\varvec{k}}_1,\sigma _1)}{ (k_1+k_2)^2+m_C^2-{\mathrm {i}}\epsilon } \right. \\&\times \left[ P^{\perp }_{\mu \kappa }({\varvec{k}})P^{\perp }_{\nu \lambda }({\varvec{k}})\right] _{{\varvec{k}}={\varvec{k}}_1+{\varvec{k}}_2} -\left[ P^{\perp }_{\mu \kappa }({\varvec{k}})P^{\perp }_{\nu \lambda }({\varvec{k}})\right] _{{\varvec{k}}={\varvec{k}}_1-{\varvec{p}}_1}\\&\times \left. \frac{{\overline{u}}({\varvec{p}}_1,s_1)\Gamma ^{\mu \nu } u({\varvec{k}}_1,\sigma _1) {\overline{v}}({\varvec{k}}_2,\sigma _2)\Gamma ^{\kappa \lambda } v({\varvec{p}}_2,s_2)}{ (k_1-p_1)^2+m_C^2-{\mathrm {i}}\epsilon } \right) . \end{aligned}$$

Here the *u* and *v* spinors are normalized such that

28 $$\begin{aligned} \sum _s u({\varvec{p}},s){\overline{u}}({\varvec{p}},s)= & {} m_e-\gamma \cdot p, \end{aligned}$$

29 $$\begin{aligned} \sum _s v({\varvec{p}},s){\overline{v}}({\varvec{p}},s)= & {} -\,m_e-\gamma \cdot p. \end{aligned}$$

## Constraints on antisymmetric tensors from Bhabha scattering

Limits on deviations from Standard Model Bhabha scattering are reported in Refs. [23–28] for energies $14\,{\mathrm {GeV}}\le \sqrt{s}\le 136.23\,{\mathrm {GeV}}$. Since $m_C>1$ TeV, the correction (25) to Bhabha scattering depends only on the product $M_e m_C$ up to corrections of less than $2\%$ in the energy range considered here. Furthermore, due to $m_e\ll \sqrt{s}$ the contribution from the dipole coupling depends only on $a_m^2+a_e^2$. Therefore we report limits on ${\tilde{M}}_e m_C$ where ${\tilde{M}}_e = M_e/\sqrt{a_m^2+a_e^2}$.

We used 11 data sets published in Refs. [34–39] and tabulated the statistical/systematic errors as a fraction of the measured cross section. To obtain lower bounds on ${\tilde{M}}_em_C$, we find the required value of the product such that the analytically obtained correction ratio drops below the reported error fractions. Since measurements of Bhabha scattering cross sections have never detected deviations from the Standard Model, this is tantamount to forcing the KR corrections to be smaller than the error bars in experimental data. Our results are tabulated in Table 1.

**Table 1:** Lower bounds on ${\tilde{M}}_e m_C$

Refs.	$\sqrt{s}$	Bound on ${\tilde{M}}_e m_C$
	GeV	$10^4\,{\mathrm {GeV}}^2$
[34]	34.5	5.4
[35]	14	1.5
[35]	22	2.0
[35]	34.8	6.1
[35]	38.3	3.5
[35]	43.6	5.3
[36]	34.7	5.0
[37]	29	6.0
[38]	52	3.4
[39]	130.26	7.1
[39]	136.23	7.1

References [34–37] include both statistical and systematic uncertainties, whereas Refs. [38, 39] report statistical uncertainties on HEPData. However, the discussion in [28] shows that the systematic uncertainties are much smaller than the statistical uncertainties for the OPAL measurements.

The strongest bound turns out to be ${\tilde{M}}_em_C\ge 7.1\times 10^4\, {\mathrm {GeV}}^2$ from the 130.26 GeV and 136.23 GeV measurements of OPAL [28]. If instead we only consider datasets where both statistical and systematic errors for cross section measurements were added in quadrature and reported, the strongest bound for ${\tilde{M}}_em_C$ is ${\tilde{M}}_em_C\ge 6.1\times 10^4\, {\mathrm {GeV}}^2$ from the 34.8 GeV measurements of TASSO [24].

## Conclusions

String theory is still the most compelling framework for particle and gravitational physics beyond the Standard Model. As such, it behooves us to seek out all possible avenues to experimental tests of string theory, and the existence of fundamental antisymmetric tensor fields is a unique prediction of string theory that should be tested at future facilities.

The clean initial states at lepton colliders will help to push the precision frontier in particle physics, and as a first study into signatures of antisymmetric tensors in Møller or Bhabha scattering at colliders, we report constraints from published data of previous experiments. We assume $m_C>1$ TeV from the absence of BSM resonances at the LHC and find the strongest constraint ${\tilde{M}}_e m_C\ge 7.1\times 10^4\, {\mathrm {GeV}}^2$ from data published by OPAL at $\sqrt{s}=130.26$ GeV and $\sqrt{s}=136.23$ GeV.

## Data Availability

This manuscript has no associated data or the data will not be deposited. [Authors’ comment: We did not generate new experimental data as part of this investigation. Data used in this paper are publicly available in Refs. [34–39]. We thank the Collaborations for having made their data available on HEPData.]

## Footnotes

1 As noted in [19], the Kalb–Ramond framework for string interactions can be generalized to the case of *N* open string species with boundary charges $g_{s,I}$, $1\le I\le N$, and corresponding boundary gauge fields ${\mathcal {B}}_{I,\mu }$. The KR gauge invariant antisymmetric tensor field in this case is

10 $$\begin{aligned} C_{\mu \nu }={\mathcal {C}}_{\mu \nu }-\frac{1}{\mu _s}\sum _I g_{s,I}{\mathcal {B}}_{I,\mu \nu }. \end{aligned}$$

A single ${\mathcal {B}}_\mu$ can be gauged away through a KR gauge transformation, but $N-1$ boundary gauge fields will always remain.

2 Since $\sqrt{s}\ll m_C$ for the experiments considered here, the parameter that makes the correction (25) small compared to Standard Model cross sections is actually $\alpha _S v_h^2 s/M_e^2 m_C^2$.
